# Development and validation of an artificial intelligence model for basal cell carcinoma detection and section completeness assessment in Mohs micrographic surgery in an Asian cohort

**DOI:** 10.1016/j.jdin.2026.08.010

**Published:** 2026-09-04

**Authors:** Li Yang Loo, Wen-Bin Luo, Isaac Hong-Kei Lam, Seow Ye Heng, Fan Foon Cheo, John Huang, Shang Wei Song, Terrence Yeoh, Fung Xue Feng, Siti Nuruljannah Baharudin, Li Yan Khor, Chee Leong Cheng, Po Yin Tang, Choon Chiat Oh

**Affiliations:** a Department of Dermatology, Singapore General Hospital, Singapore; b Department of Anatomical Pathology, Singapore General Hospital, Singapore; c Duke-National University of Singapore Academic Medical Centre, Singapore; d AI Singapore (ex-AI Apprenticeship Programme), Singapore; e AI Singapore, Singapore

**Keywords:** artificial intelligence, basal cell carcinoma, convolutional neural network, dermatopathology, Mohs micrographic surgery, tissue completeness, tumor detection

## Abstract

**Background:**

Mohs micrographic surgery for basal cell carcinoma (BCC) requires rapid intraoperative histologic interpretation. This is labor-intensive and incomplete tissue sections compromise margin assessment. Artificial intelligence has shown promise in BCC detection but section completeness assessment remains underexplored.

**Objective:**

Develop and validate an artificial intelligence model for BCC detection and section completeness assessment on Mohs frozen-section histology.

**Methods:**

We conducted a retrospective single-center cohort study using histology slides from 130 patients who underwent Mohs surgery for BCC from 2017 to 2025. Whole slides were digitized and annotated. A convolutional neural network model was trained to detect BCC and classify section completeness. Model performance assessment with standard performance metrics and region-of-interest evaluation was performed.

**Results:**

The BCC detection model achieved accuracy 0.993, sensitivity 0.915, specificity 0.996, precision 0.889, F1-score 0.902 at the tile level, with comparable region-of-interest sensitivity (0.910). The section completeness model achieved accuracy 0.890, sensitivity 0.948, specificity 0.524, precision 0.927, and F1-score 0.937.

**Limitations:**

Single-center retrospective design, limited representation of rare BCC subtypes, and no direct comparison with human experts.

**Conclusion:**

Although the specificity for section completeness was low, further research and additional model training may enable development of a model with improved performance.

**Capsule Summary**

• We demonstrate that artificial intelligence accurately detects basal cell carcinoma in Mohs surgery histology in an Asian cohort, while directly assessing section completeness.
• Artificial intelligence-assisted analysis not only detects basal cell carcinoma but can provide output on incomplete sections, improving workflow efficiency and reducing diagnostic oversight.

## Introduction

Nonmelanoma skin cancer is the most commonly diagnosed skin cancer worldwide.^1^ Basal Cell Carcinoma (BCC) incidence is rising 4% to 8% annually.^2^^,^^3^ Aging populations are expected to drive continued increases, placing increasing burden on dermatologic services.^4^

Mohs micrographic surgery (MMS) is the gold standard for treating BCC at high-risk locations like the face, offering precise margin control and high cure rates.^5^ This requires accurate intraoperative histologic assessment. Section completeness is a prerequisite for reliable margin evaluation. The existing workflow is time-consuming. Each surgical stage requires real-time processing, sectioning, staining, and histologic interpretation before further excision decisions are made.^6^

Recent advances in artificial intelligence (AI) have demonstrated high diagnostic accuracy for BCC detection on histopathology.^7^ Convolutional Neural Networks, designed to analyse grid-like data through hierarchical spatial feature learning have shown promise in medical imaging applications.^8^ Graph Neural Networks extend learning to graphical data, where relationships between entities are modelled to capture contextual patterns not easily represented in conventional image-based models.^9^

Existing studies focused on tumor detection, yet only one has reported on section completeness—a critical determinant of interpretability in MMS.^10^ To our knowledge, no study has provided quantitative AI-based output on MMS section completeness. We aimed to develop an AI model for simultaneous BCC detection and section completeness assessment.

## Methods

### Study design

A retrospective, single-center cohort study was conducted at the Singapore General Hospital Department of Dermatology with SingHealth Institutional Review Board approval (IRB 2020/2036). Whole slide images (WSIs) and clinical data from 130 patients who underwent MMS for BCC from January 2017 to December 2025 were included. A Convolutional Neural Network-based AI model was developed for 2 tasks:

1. BCC detection: Classification of slide regions (patches) into “tumor-positive” or “tumor-negative.”
2. Section completeness assessment: Classification of sections as “complete” or “incomplete” based on the presence of holes, tears, or processing artifacts (folds).

### Power analysis

Sample size calculations were based on a previous study reporting area under the curve (AUC) of the receiver operating characteristic curve of 0.975 (standard deviation 0.027) for BCC detection and 0.923 (standard deviation 0.0629) for section completeness assessment.^10^ To achieve target AUC of 0.98 and 0.935 respectively, at alpha 0.05 and 90% power, a minimum of 306 sections for BCC detection and 289 sections for completeness assessment were required.

### Dataset generation

Archived histology slides from 130 BCC MMS cases were digitized into WSIs using a high-resolution slide scanner (PHILIPS SG300) at 40x magnification with a resolution of 0.25um per pixel and de-identified. Each MMS operation produced an average of 6 representative WSIs for each patient, each containing 2 to 3 sections. De-identified patient data including age, sex, tumour subtype, and anatomical site were collected. Each WSI was annotated by 2 dermatopathologists using OMERO (an open-source platform with password protection on a secure server), with a third reviewer resolving discrepancies. To ensure precise segmentation, 2 authors (L.W.B., T.P.Y.) outlined BCC regions and annotated nonmalignant structures, including hair follicles, sweat ducts, and sebaceous glands.

Sections were complete if they fulfilled these criteria, verified by 2 authors (L.L.Y., O.C.C.): <10% epidermis missing or folded, <5% area occupied by holes and holes were <1.5 mm (Representative examples in Fig 1). Discrepancies were resolved through discussion.

**Fig 1:**
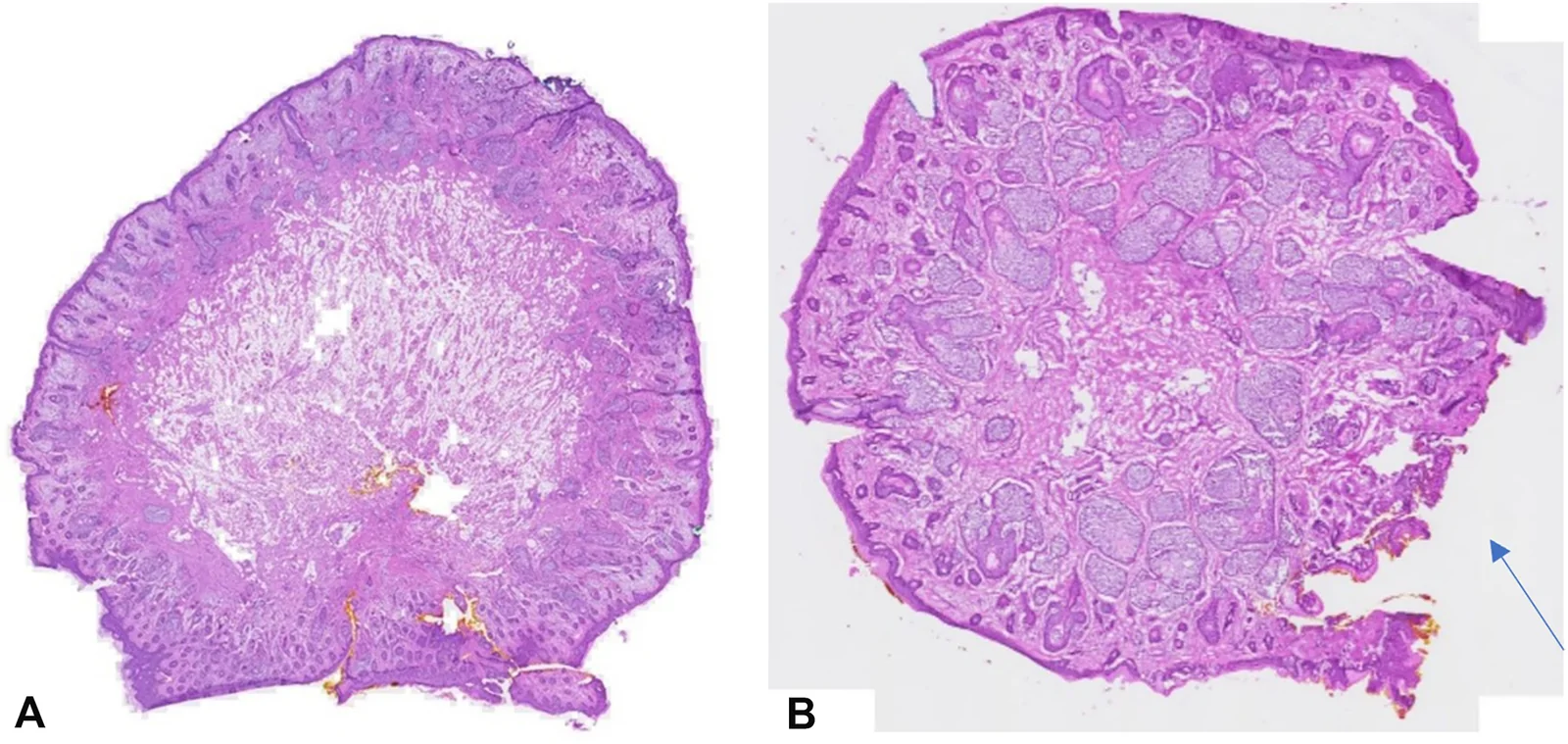
Representative examples of complete and incomplete slides. Stain: hematoxylin and eosin. **A,** A complete section, **(B)** an incomplete section (*blue arrow* indicating incomplete area).

A WSI was classified as majorly out-of-focus when more than half the section is not in focus. These were excluded from the BCC detection dataset. For section completeness classification model development, curette slides from the first stage of MMS were excluded as they are invariably incomplete.

### Preprocessing and image augmentation

Each WSI was tiled at 5× magnification into the overlapping patches (224 × 224 pixels). Tiles containing insufficient tissue were excluded using intensity thresholding. Reinhard stain normalization was applied using pooled tissue-level statistics. For the section completeness model, images of sections were extracted. A stratified group split ensured that WSIs from the same patient were confined to either the training, validation or test set in an approximately 80:10:10 ratio. Datasets were stratified to ensure representation of tumor subtypes and artifact types while maximizing training data. Model development was performed using the training and validation set, with final evaluation performed on the held-out test set.

Data augmentation was applied only to the training dataset. Rotations and flips (8 square symmetries; *P* = 1.0),^11^ color jitter (brightness, contrast, saturation and hue randomly perturbed; *P* = 1.0)^12^ and defocus blur (simulating out-of-focus regions and optical variation; *P* = .5)^13^ were applied to training datasets of both tasks.

Grid dropout (random masking of grid-like regions to encourage spatial robustness; *P* = .5)^14^ was used only for BCC detection model training. Grid Distortion (structured spatial distortions to simulate artifacts; *P* = .85) and Elastic Transform (smooth, nonlinear spatial warping to mimic tissue distortion during slide preparation; *P* = .85)^15^ were used only for section completeness classification model training as a regularization technique to improve the model’s robustness to tissue shape and structure variability.

### Model development

The deployed models used the ConvNeXt-Tiny backbone pretrained using DINOv3 (Meta Platforms, Inc) self-supervised learning on the LVD-1689M dataset.^16^^,^^17^ To tackle class imbalance,^18^ a positive class weight of 1.1 was applied to the binary cross-entropy loss during model training. A weighted random sampler was implemented, using max-normalized inverse class frequency. To reduce overfitting, dropout (0.5) was applied in the classifier head, weight decay (0.01) was used in the AdamW optimizer (learning rate 1e-5), gradient norm clipping was applied at 0.1 and 0.5, and early stopping monitored validation loss. Full 32 bit precision is used throughout. BCC detection and slide completeness models used batch sizes of 99 and 64 respectively. The validation set was used for hyperparameter tuning and model-configuration selection. Within each training run, early stopping and checkpoint selection were based on validation loss, with patience values of 50 epochs for BCC detection and 20 epochs for section completeness assessment. Following configuration selection, the final models were retrained on the combined training and validation sets.

### Performance evaluation

Accuracy, sensitivity, specificity, precision, F1-score, AUC-receiver operating characteristic curve and AUC of the precision recall curve (PRC) was calculated for tile-level BCC detection (probability threshold 0.5) and for section completeness as tumor-positive tiles and complete slides were outnumbered by tumor-negative tiles and incomplete slides. Incomplete slides were the clinically important result. 95% confidence intervals were calculated using patient-level clustered resampling (10,000 bootstrap resamples with a fixed random seed; each bootstrap sample comprised all tiles for BCC detection and all sections for completeness assessment corresponding to the resampled patient) as the training, validation, and test split was performed at the patient level.

A secondary qualitative review of false-positive tiles was performed to characterize model errors. False-positives are tiles labelled as BCC-negative with predicted probability ≥0.5, while false-negatives are tiles labelled BCC-positive with predicted probability ≤0.5. A patient-stratified random sample of 100 false-positive tiles was reviewed, with each patient’s contribution proportional to their share of the false-positive pool. Sampling was performed without replacement.

As tile-level classification of BCC presence does not reflect how clinicians interpret model outputs in practice, region-of-interest (ROI) detection (identification of BCC location) was evaluated at section-level.^19^ True positive ROIs are based on human polygon annotations, whereas false positive regions are generated using 8-connectivity analysis on predicted regions. For ROI-level evaluation, specificity requires true negative counts which are not well-defined in the ROI-level framework.^20^ There are no negative ROIs for calculating true negatives. Instead, false-positive predicted regions (predicted ROIs occurring outside ground-truth ROIs) were calculated.

### Model output

An application workflow was developed. The back-end architecture integrates AI models for BCC detection and section completeness assessment (Fig 2, *A*). After front-end input, the model completes its inference and results are displayed on the front-end. If BCC is detected, the specific tile locations are automatically mapped onto the WSI for tumor localization and margin evaluation (Fig 2, *B*). If no BCC is identified, the model classifies section completeness. (Fig 2, *C*).

**Fig 2:**
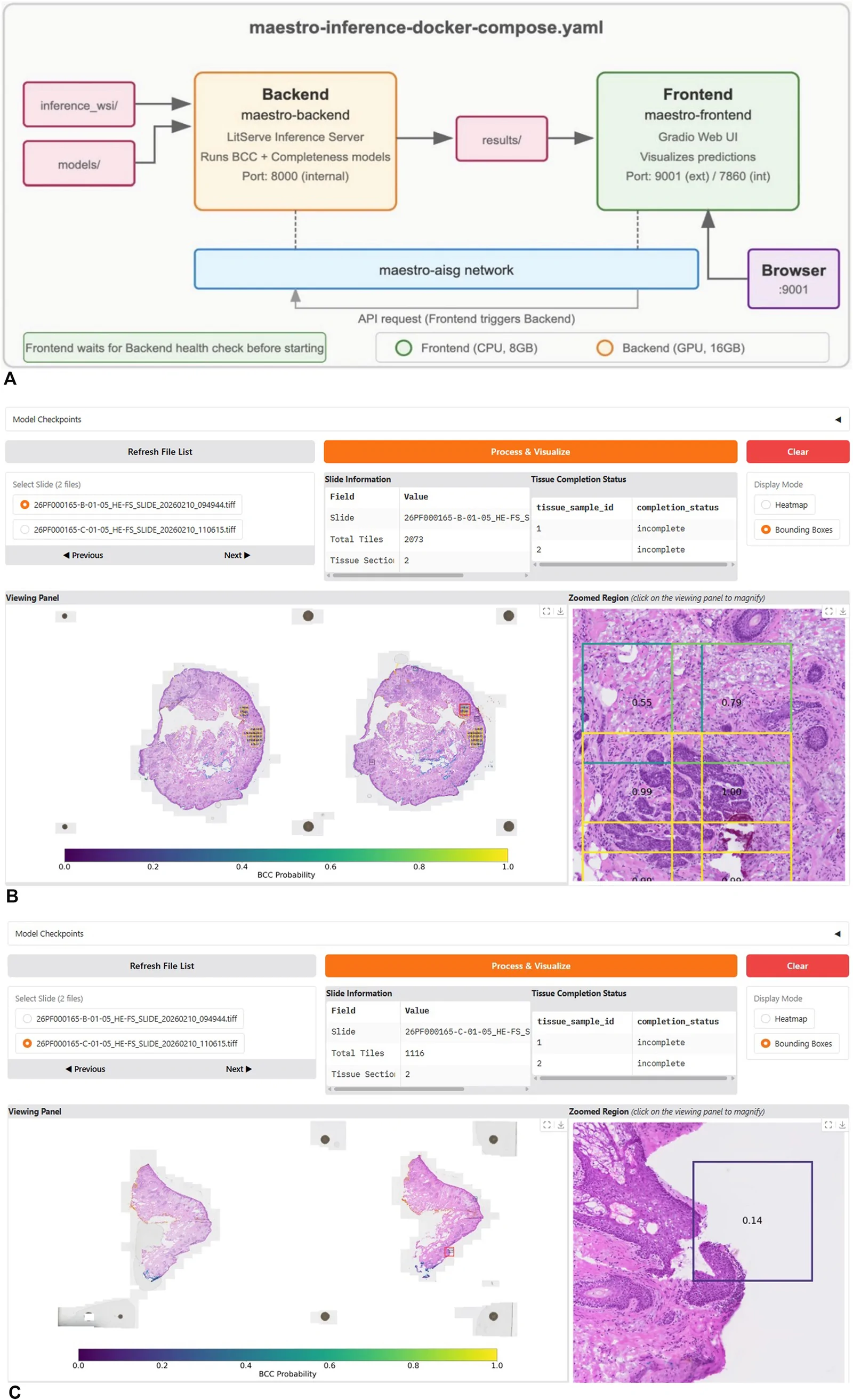
Application workflow and example output. **A,** Flow chart of data process. The Mohs surgeon initiates the inference request via the front-end interface. Following back-end processing, the model outputs are relayed back to the front end for clinical review and validation. **B,** Example outputs of BCC detection with heatmap and bounding box. The section viewed is selected in the *top left panel*, with the *top middle* panel showing the number of tiles in the section and number of sections, along with assessment of completeness of each section. At low power on the *bottom left panel*, AI-predicted BCC areas are highlighted by bounding boxes, with the color of boxes dependent on the probability of BCC. *Red boxes* at the low-power view indicate ROI where the surgeon is reviewing at high-power view. At higher power on the *bottom right panel*, the predicted probability of BCC is displayed within the yellow bounding boxes. **C,** Example outputs of tissue completeness with bounding boxes. At low-power view on the *bottom left*, AI-predicted areas of section incompleteness are highlighted by bounding boxes, with the color of the boxes indicating the probability of section incompleteness. At the high-power view on the bottom right panel, the predicted probability of tissue completeness is displayed within the bounding box. The lower the probability, the more likely the tissue is incomplete. *BCC*, Basal cell carcinoma; *ROI*, region of interest.

## Results

A total of 130 patients with histology-proven BCC were included in the study. 50% were male. 117 (90.0%) were Chinese, 7 (5.4%) were Caucasian, 3 (2.3%) were Malay, while 3 (2.3%) were of other races. Mean age was 72.8 years (standard deviation 10.9). A majority of tumors were located on the nose (*n* = 58, 44.6%), cheek (*n* = 28, 21.5%) and periorbital (*n* = 12, 9.2%) areas. Patient characteristics are presented in Table I.

**Table I:** Patient and tumor characteristics

Age	
Mean (SD)	72.8
Minimum	34
Maximum	96
Sex, *n* (%)	
Male	65 (50.0%)
Female	65 (50.0%)
Race, *n* (%)	
Chinese	117 (90.0%)
Caucasian	7 (5.4%)
Malay	3 (2.3%)
Other	3 (2.3%)
Tumor location, *n* (%)	
Nose (including Ala, Tip, Side Wall, Bridge, and Dorsum)	58 (44.6%)
Cheek (including nasolabial fold)	28 (21.5%)
Periorbital (including eyelids, canthus, and eyebrows)	12 (9.2%)
Forehead (including Glabella)	8 (6.2%)
Temple	8 (6.2%)
Lip	7 (5.4%)
Scalp	4 (3.1%)
Ear	3 (2.3%)
Other	2 (1.5%)
Subtypes	
Nodular	124 (95.4%)
Infiltrative	5 (3.8%)
Infundibulocystic	1 (0.8%)

Other races: 1 Indian, 1 Indonesian, 1 Vietnamese.

*SD*, Standard deviation.

In total, 734 WSIs were included. 66 majorly out-of-focus WSIs were excluded from the BCC detection dataset, leaving 668 WSIs for BCC detection model development. One hundred twelve curette slides were excluded from the section completeness dataset, leaving 622 WSIs for slide completeness assessment model development.

A total of 1,153,144 tiles and 1540 tissue sections were generated during preprocessing. For the BCC detection task, the training and validation sets for model development contained 1,028,525 tiles (3.77% BCC-positive). The test set contained 124,619 tiles (3.39% BCC-positive). For the section completeness task, the training and validation sets for model development contained 1385 tissue sections, (13.36% labelled complete). The test set contained 155 tissue sections (13.55% labelled complete). This is presented in Table II.

**Table II:** Number of tiles and sections used for BCC detection and section completeness classification model training, validation, and test sets

	Total	Model development (training and validation sets)	Test
Cases	130	117	13
Tiles (for BCC detection) generated from 668 WSIs included	1,153,144	1,028,525	124,619
Positive	42,997 (3.7%)	38,775 (3.8%)	4222 (3.4%)
Negative	1,110,147 (96.3%)	989,750 (96.2%)	120,397 (96.6%)
Sections (for section completeness classification) generated from 622 WSIs included	1540	1385	155
Complete	206 (13.4%)	185 (13.4%)	21 (13.5%)
Incomplete	1334 (86.6%)	1200 (86.6%)	134 (86.5%)

*BCC*, Basal cell carcinoma; *WSI*, whole slide image.

### BCC detection

A total of 119,914 tiles were correctly classified as tumor-negative (true-negatives) and 3862 tiles were correctly identified as tumor-positive (true-positives). There were 483 false-positive predictions and 360 false-negative predictions (Table III).

**Table III:** Confusion matrix for BCC detection at tile level

	Predicted label		
	No BCC	BCC	Total
True label			
No BCC	True negative 119,914 (99.6%)	False positive 483 (0.4%)	120,397
BCC	False negative 360 (8.5%)	True positive 3862 (91.5%)	4222
Total	120,274	4,345	124,619

*BCC*, Basal cell carcinoma.

Classification accuracy was 0.993 (95% CI 0.993-0.994). The BCC detection model achieved a sensitivity of 0.915 (95% CI 0.873-0.955), specificity of 0.996 (95% CI 0.994-0.998), precision (positive predictive value) of 0.889 (95% CI 0.775-0.944), negative predictive value 0.997 (95% CI 0.994-0.999) and F1-score of 0.902 (95% CI 0.833-0.941). AUC-receiver operating characteristic curve (Fig 3, *A*) for BCC detection was 0.992 (95% CI 0.987-0.996) and AUC-PRC (Fig 3, *B*) was 0.957 (95% CI 0.917-0.980). Review of false positives showed that 26% arose due to BCC mimickers (stratum basalis, hair follicles, and sweat ducts), 37% arose from inflammatory infiltrate, 35% arose from fibrous tissue and keratin, while 2% were due to slide or scanning artifacts.

**Fig 3:**
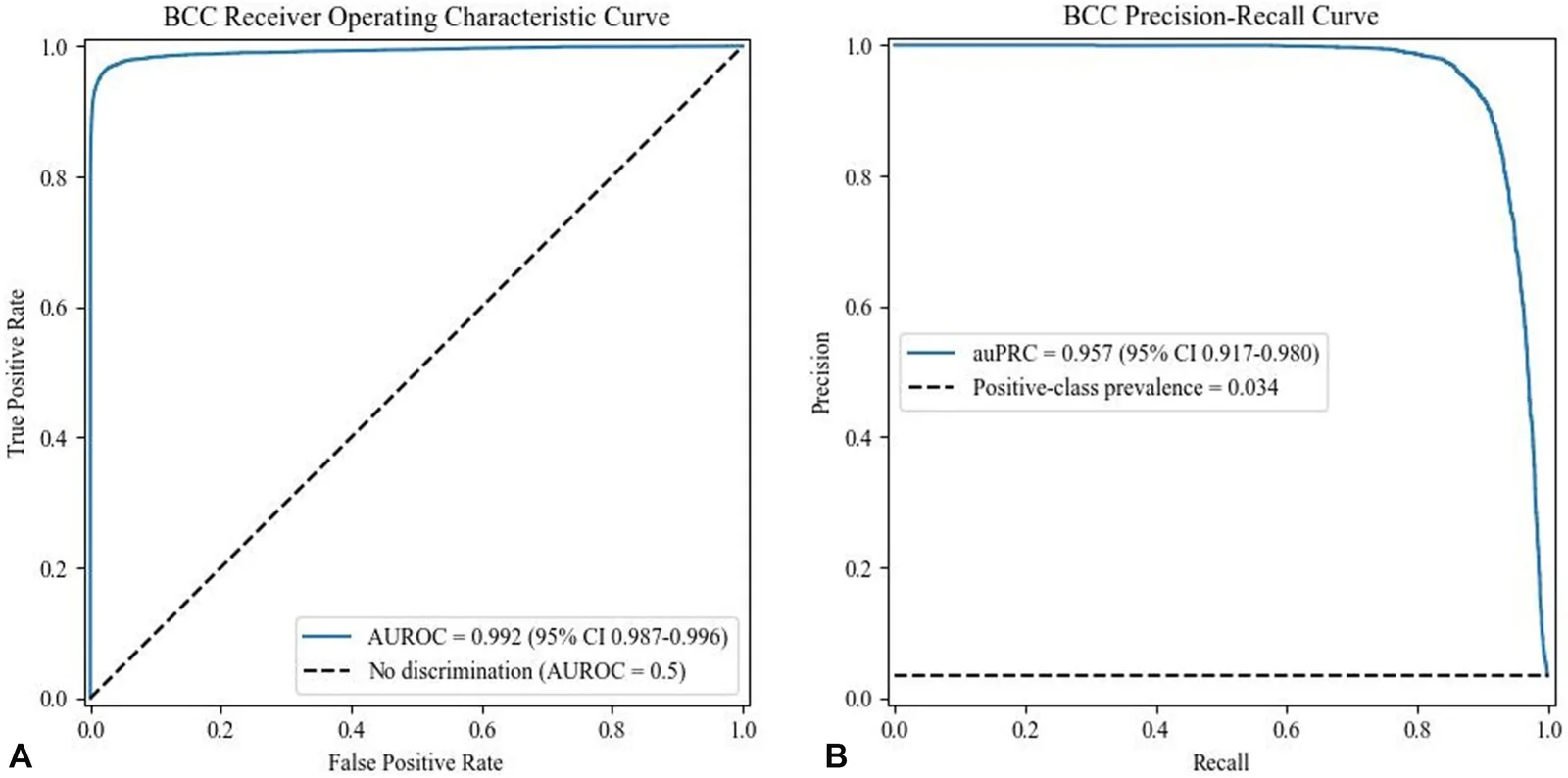
Overall assessment of BCC detection. **A,** Receiver operating characteristic curve (AUC 0.992, 95% CI 0.987-0.996), **(B)** Precision recall curve (AUC 0.957, 95% CI 0.917-9-0.980) . *AUC*, Area under the curve; *BCC*, basal cell carcinoma.

The ROI-level evaluation performed similarly to the tile level, with a sensitivity of 0.910 (95% CI 0.876-0.957) and 336 false-positive regions identified across 155 test sections (average of 2.17 false positive regions per section).

### Section completeness

For section completeness classification, 127 sections were correctly classified as incomplete (true-positives) while 11 were correctly classified as complete (true-negatives). The model produced 7 false-negative predictions and 10 false-positive predictions (Table IV). The false reassurance rate (proportion of truly incomplete sections incorrectly classified as complete) was 0.052 (95% CI 0.026-0.104).

**Table IV:** Confusion matrix of test predictions for section completeness classification

	Predicted label		
	Complete	Incomplete	Total
True label			
Complete	True negative 11 (52.4%)	False positive 10 (47.6%)	21
Incomplete	False negative 7 (5.2%)	True positive 127 (94.8%)	134
Total	18	137	155

The section completeness classification model achieved accuracy of 0.890 (95% CI 0.841-0.940), sensitivity of 0.948 (95% CI 0.880-0.992), specificity of 0.524 (95% CI 0.250-0.857), precision (positive predictive value) of 0.927 (95% CI 0.859–0.985), negative predictive value of 0.611 (95% CI 0.300-0.909), F1-score of 0.937 (95% CI 0.888-0.976), with AUC-ROC 0.923 (95% CI 0.863-0.980) (Fig 4, *A*) and AUC-PRC 0.988 (95% CI 0.972-0.998) (Fig 4, *B*).

**Fig 4:**
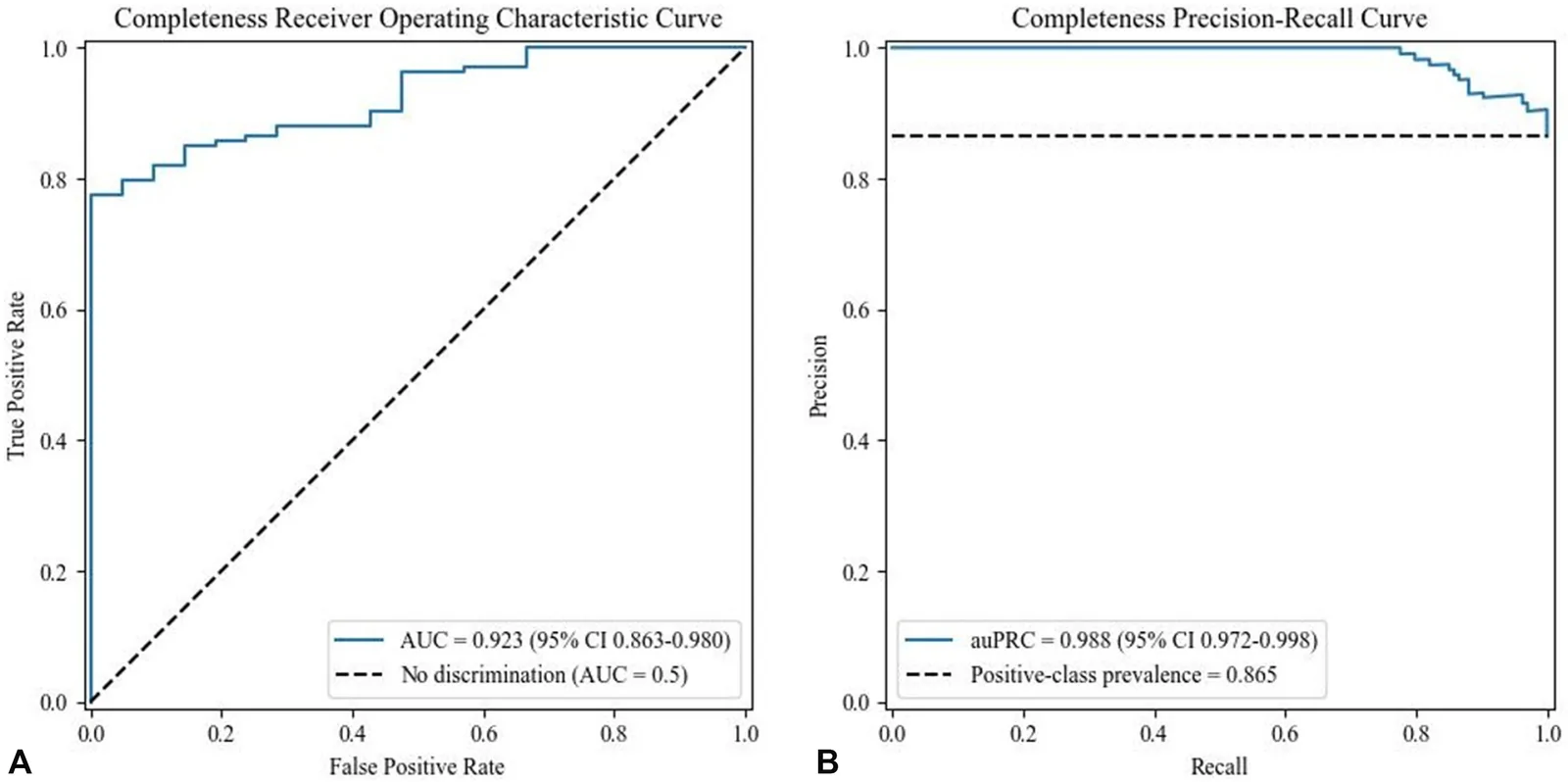
Overall assessment of section completeness classification. **A,** Receiver operating characteristic curve (AUC 0.923, 95% CI 0.863-0.980) and **(B)** Precision recall curve (AUC 0.988, 95% CI 0.972-0.998). *AUC*, Area under the curve.

## Discussion

In this study, we developed and validated an AI model for both BCC detection and tissue section completeness assessment in MMS frozen sections at a single center. Previous studies were conducted in America,^10^^,^^21^^,^^22^ France and Turkiye.^23^^,^^24^ To our knowledge, we are the first to address an Asian cohort. Our model demonstrated high performance for tile-level tumor detection, with clinically meaningful ROI localization. It predicts section completeness probability, an underexplored requirement for margin interpretation during MMS.

### Dataset, annotation, and model selection

Our dataset comprised high-resolution WSIs annotated to delineate precise contours of BCC for deep learning, providing advantages for detection and localization.^10^ Previous studies identified BCC in WSI, yielding a binary classification of tumor presence.^21^^,^^22^ This leaves tumor localization to the dermatopathologist, limiting intraoperative utility.

A deep learning model capable of localization guides the pathologist to ROI to confirm the diagnosis efficiently. While Bonnefille et al employed a patch-based EfficientNet for classification and localization,^23^ Yilmaz et al utilized a dual-architecture approach comprising ResNet-101 for classification and U-Net for segmentation.^24^ ConvNeXt-Tiny was selected for our model backbone to balance between capacity and computational efficiency. As a modernized ResNet-style convolutional architecture that incorporates vision transformer design principles while retaining a convolutional structure,^16^ it offered a contemporary alternative to traditional backbones such as ResNet without the computational burden of larger transformer-based models. This was practical given the high tile volume inherent to patch-based WSI inference.

### BCC detection

To our knowledge, ROI-level evaluation has not previously been reported for BCC. Unlike tile-level assessment, ROI-level evaluation more closely approximates clinical practice as clinicians evaluate contiguous tumor regions rather than isolated image patches.^19^^,^^25^ Our model’s ROI sensitivity was 0.910. The average of 2.17 false-positive regions per section may represent an acceptable screening burden in clinical practice. Tile-level performance was comparable to existing studies for sensitivity, specificity, precision, F1-score, and AUC-ROC.^10^^,^^23^^,^^24^^,^^26^

Our dataset demonstrated substantial class imbalance (96% of tiles tumor-negative and 86% of sections incomplete). ROC curves may overestimate performance in such settings as true negatives dominate classification metrics,^27^ thus AUC-PRC was reported. Our model’s AUC-PRC of 0.957 substantially exceeded the expected baseline of 0.034 for a random classifier at the observed positive tile prevalence of 3.4%. To our knowledge, explicit reporting of PRC in MMS frozen-section AI literature remains limited, despite the imbalanced nature of these datasets.

False-positives in AI-based BCC detection are attributed to biological mimickers (hair follicles, sebaceous glands, eccrine ducts, actinic keratosis, seborrheic keratosis, Bowen’s disease, nevi, inflammatory cells)^21^^,^^23^^,^^26^^,^^28^, ^29^, ^30^ and technical artifacts (out-of-focus slides, folds, staining variations, air bubbles).^21^^,^^26^^,^^29^ In our cohort, 98% of false-positive tiles arose from biological mimickers with technical artifacts only accounting for 2%.

False-negatives in BCC detection arise from small tumor foci compared to specimen size or specific growth patterns (micronodular or infiltrative BCC).^23^ Ground truth inaccuracies also cause classification errors.^26^ We carefully annotated tiny BCC clusters and assured WSI scanning quality to maximize ground-truth accuracy. In our study, many false-negatives did not contain definite BCC but fell within broadly annotated BCC regions, suggesting that tile-level label noise from coarse annotations may account for a proportion of false-negatives rather than true model failures.

### Tissue section completeness assessment

Existing AI studies in MMS have largely focused on tumor detection.^21^^,^^22^^,^^24^^,^^26^^,^^29^^,^^31^ However, accurate margin interpretation first requires confirmation of tissue section adequacy.^32^ Incomplete epidermal representation (tears, folds), holes, or missing deep margins may render a tumor-negative interpretation unreliable, necessitating re-sectioning. To our knowledge, limited work has evaluated AI-driven section completeness assessment in MMS. Unlike Levy et al, whose model generated heatmaps of potential tears and holes,^10^ our model directly generates section completeness probability, an interpretable measure more aligned with intraoperative decision-making. Our model also accounts for incompleteness beyond tears and holes, like missing epidermis or folds. Its high true-positive rate could enable rapid identification of incomplete sections, possibly prompting earlier re-sectioning and enhancing surgical workflow.^33^ However, this has yet to be evaluated in this study.

False-negatives correlated with noncircular geometries, most commonly encountered at specific anatomical sites with naturally irregular contours (eg eyelid). False-positives resulted from tissue processing artifacts (eg minor clefts), which were misidentified as section fragmentation.

## Limitations

Our single-center study is limited in rare BCC subtypes and mimicker lesions, potentially affecting generalizability. Due to a small sample size, section completeness assessment requires further refinement, and remains susceptible to misclassification from irregular specimen geometries and tissue-processing artifacts. Consequently, human validation of AI-generated predictions remains critical. Further work is planned to evaluate specimens from additional centers to refine the model.

Color variations and suboptimal WSI quality may compromise BCC detection accuracy. Future efforts will incorporate artifact segmentation tools into the preprocessing pipeline as a quality control measure. This ensures only high-fidelity images proceed to final inference. While external validation and comparison with human readers were not performed in this study, our model demonstrated competitive performance when benchmarked against previously reported metrics. Validation on external databases is ongoing.

The impact of this AI model on clinical practice has yet to be measured. Prospective evaluation in clinical settings is underway and may identify real-world implementation challenges for troubleshooting, before allowing for AI-integrated workflow optimization.

## Conclusion

This study demonstrates the feasibility of AI for simultaneous BCC detection and section completeness assessment in MMS frozen sections within an Asian cohort at a single center. Our model achieved competitive performance for tumor detection. It provides a novel, interpretable probability output for section completeness assessment – an underexplored yet clinically critical component of MMS.

### Declaration of generative AI and AI-assisted technologies in the writing process

None.

## Conflicts of interest

None disclosed.
